# Features of drug addiction treatment programs in Atlantic Canada that help (or not) with access and retention: A qualitative study

**DOI:** 10.1371/journal.pone.0328524

**Published:** 2025-08-04

**Authors:** Holly Mathias, Lois A. Jackson, Jane A. Buxton, Anik Dubé, Niki Kiepek, Jo-Ann MacDonald, Fiona Martin, Jen Smith

**Affiliations:** 1 School of Public Health, University of Alberta, Edmonton, Alberta, Canada; 2 School of Health and Human Performance, Dalhousie University, Halifax, Nova Scotia, Canada; 3 School of Population and Public Health, University of British Columbia, Vancouver, British Columbia, Canada; 4 Faculty of Health Sciences and Community Services, School of Nursing, Université de Moncton, Moncton, New Brunswick, Canada; 5 School of Occupational Therapy, Dalhousie University, Halifax, Nova Scotia, Canada; 6 Faculty of Nursing, University of Prince Edward Island, Charlottetown, Prince Edward Island, Canada; 7 Department of Sociology and Social Anthropology, Dalhousie University, Halifax, Nova Scotia, Canada; 8 Eastern Health, St. John’s, Newfoundland, Canada; Government of Nova Scotia, CANADA

## Abstract

Access to government-funded addiction treatment programs can reduce harms experienced by people who use substances (PWUS). There is some research on what features (e.g., policies and practices) of treatment programs help or do not help with access; however, not much is known about program directors’ and physicians’ perspectives of these features in Atlantic Canada. One-on-one semi-structured qualitative interviews were conducted with program directors and physicians working in government-funded addiction treatment programs in Atlantic Canada in 2021. Interview questions focused on perspectives of program features that helped or not with access and retention, including perspectives on changes due to COVID-19. Data were coded and analyzed using grounded theory techniques to develop themes and subthemes. Fourteen individuals were interviewed. They identified several features that helped (e.g., quick access) or did not help (e.g., wait times) with access and retention. Participants shared some features that changed due to COVID-19, including some that helped (e.g., virtual services) and did not (e.g., limited program spaces). Participants suggested changes that could support access and retention, including better linkages to mental health supports. This paper highlights program directors’ and physicians’ perspectives on how program features inform access and retention in Atlantic Canada. Findings on changes made during COVID-19 point to the need to maintain the changes that were helpful and implement additional changes to better support access for more PWUS. To support the implementation and sustainability of these changes, more resources must be invested.

## Introduction

Harmful substance use is a pressing issue in many parts of the world, including Canada [1–5]. People who inject or smoke drugs are at risk of health and social harms, such as risks of HIV, bacterial infections, and the risk of arrest [6–8]. Between January 2016 and March 2024, more than 47,000 Canadians died from ‘apparent opioid toxicity’ [2]. Most of these deaths occurred in the provinces of British Columbia, Alberta, and Ontario; however, all provinces and territories have been affected. During this period, there were 1144 deaths in Atlantic Canada (a region comprised of the four most easternly provinces) [2].

Ensuring access to government-funded drug treatment programs is one strategy that, in combination with a comprehensive array of harm reduction programs and policies, helps to reduce harms. Government-funded treatment programs vary across Canadian provinces, but typically include withdrawal management services (i.e., detoxification) and opioid agonist treatment (OAT) (e.g., methadone, buprenorphine) programs [8,9]. For people seeking treatment, detoxification programs, referred to as ‘detox’ throughout this paper, are often the first step [10]. Detox programs vary in terms of the length of time people who use substances (PWUS) can stay (e.g., a few days to several weeks), program content (e.g., counselling and educational supports), and settings (e.g., inpatient or outpatient) [9,11]. OAT programmes are the “gold standard” of opioid use disorder treatment [12]. They also vary in terms of length or PWUS involvement in the program (e.g., months or years), and settings (e.g., clinics) [12,13].

It is important to understand how the features of treatment programs (e.g., policies and practices) may help or not help with access to and retention in treatment. Many features that are not helpful have been identified in the Canadian context and elsewhere, such as wait times, challenges securing transportation and childcare, stigma, and negative interactions with treatment providers [14–19]. Some program features that help with access to and retention in treatment have also been identified in the literature, including program location, hours of operation, and supportive staff [20–22].

We conducted a three-phase qualitative research study exploring features of treatment programs in Atlantic Canada that were helpful or not helpful for PWUS when they were trying to access and stay in treatment to better understand access in this setting. This paper presents findings from Phase 3 which involved interviews with directors and physicians of government-funded treatment programs. We collected data from other key informants in earlier phases. Phase 1 of our research focused on the perspectives of PWUS. They identified a range of features that were not helpful, including wait times, limited hours of operations, and programs being located far from where PWUS live [23]. PWUS also identified a few features they deemed to be helpful and thought should be expanded, such as readily available access to treatment and supportive staff [24]. In Phase 2 of the research, we asked family members/family of choice of PWUS and community-based organization staff about their perspectives on treatment program features and access and retention. These two groups also identified several features that were helpful (e.g., supportive staff) and not helpful (e.g., program location, wait times). They also provided a variety of supports to help PWUS with access such as assisting with transportation to treatment and providing access to a phone to contact a program [24].

In this paper, we present findings from the third and final phase of the study which involved interviews with directors and physicians of government-funded detox and OAT clinics in Atlantic Canada. We also provide practice and policy recommendations to address some of the key barriers to access and retention identified by participants. The perspectives of these two groups are important as they work in the programs of interest and are knowledgeable about program features that may affect access. This paper adds to the body of literature on access to and retention in treatment by providing the perspectives of directors and physicians working in treatment programs in Atlantic Canada, including insights into program changes made during COVID-19.

## Methods

Our community-based qualitative study was developed in partnership with several community knowledge users and academics from across Atlantic Canada (please see our previous work [23,24] for more details on study design and team composition). Atlantic Canada is the most easternly region of Canada comprised of four provinces (New Brunswick, Newfoundland and Labrador, Nova Scotia, and Prince Edward Island [PEI]) (population: 2.6 million) [25]. At the time of data collection, all four Atlantic provinces provided government-funded detox and OAT programs; however, it is unknown how many PWUS were accessing treatment [9]. Data from 2024 and 2025 indicates that wait times for non-urgent adult mental health and addiction treatment programs in Nova Scotia and New Brunswick range from 29 to 240 days. No publicly available data exists for PEI and Newfoundland [26,27]. The study was reviewed and approved by the following institutional research ethics boards: Eastern Health Research Proposals Approval Committee (#2020–117); Health PEI Research Ethics Board (#6008731); Health Research Ethics Authority (#20210551); Horizon Health Network (#2020–2903); Nova Scotia Health Research Ethics Board (#1025957); Université de Moncton (#2021–031); University of British Columbia Behavioural Research Ethics Board (#H20-00418); University of Ottawa (#H12-20–5694); and University of Prince Edward Island (#6008731). Our work is reported on using the Standards for Reporting Qualitative Research (SRQR) [28].

### Participants and recruitment

Individuals were eligible to participate in the study if they met the following inclusion criteria: a) employed at a government-funded treatment program as a director or physician in Atlantic Canada; and b) believed they had worked at the program long enough to be able to speak to the research questions. In some cases, directors may have also been employed as physicians within the program. Exclusion criteria included: a) working in a private treatment facility; or b) holding a position other than director or physician.

Prior to recruitment, government-funded detox and OAT programs in Atlantic Canada were identified through a review of provincial health authority websites in each of the four Atlantic provinces. From these identified programs, programs were purposively selected to provide a mix of detox and OAT programs in both urban and non-urban areas. The research coordinator (Author HM) emailed the director of each of the five programs and invited them to participate in the study. If there was no response after one week, one of the research team members (e.g., research coordinator or knowledge users who had some professional contact with the program) followed up with the program via phone or email. If the program director was unable to participate, we asked them to forward the invitation to a physician within the program. All interviews were scheduled at a mutually agreed upon time. We also relied on snowball sampling to recruit some participants. For example, we asked participants to share the invitation to participate with other program directors and physicians who might be eligible. Recruitment took place between May 1, 2021 and July 16, 2021. Twenty-four individuals were contacted from across the four Atlantic provinces and asked if they would like to participate. Fourteen individuals agreed to participate, with participants from all four provinces.

### Data collection

One-on-one semi-structured telephone interviews took place between May-July 2021. An interview guide (S1 Appendix) was developed with members of the research team, which included individuals who worked with PWUS and had some knowledge of the treatment program. The guide was piloted with one of the study knowledge users who was the director of a low-threshold OAT program and not eligible to participate in the study since they were a research team member. The interview questions aimed to explore participants’ overall perceptions of the feelings of PWUS when accessing treatment, as well as how features of government-funded treatment programs help or not help with program access and retention, with a focus on the previous two years. We asked participants to speak about their general experiences with clients, although they could also provide examples if desired. For example, when talking about access to programs, they were asked ‘Can you tell me about any program policies or practices that you perceived were helpful for your clients when trying to get in?’ and probes were used as necessary (e.g., transportation, childcare). One interview question asked about perceived changes to PWUS’ substance use while accessing or staying in treatment; however, these data are not reported here. The overall study was designed in 2018, prior to the onset of COVID-19. However, this phase of interviews took place during early 2021, therefore we amended the interview guide to include a question about the impact of COVID-19 on program features (e.g., policies and practices). These amendments were approved by the relevant institutional research ethics boards. Select sociodemographic information (e.g., gender identity, age range) was also collected to contextualize the research findings.

The research coordinator, a woman with several years of qualitative research training and experience, conducted all interviews. At the beginning of the interview, the research coordinator reviewed the informed consent form and answered any questions before obtaining verbal consent. Consent was documented by the interviewer signing and dating the informed consent form. This process was approved by the institutional ethics boards. All participants were offered an honorarium in the form of a $20 CAD e-gift card that was sent via email after the interview. All interviews were audio recorded with the permission of the participant. The audio recordings were transferred via a secure institutional file transfer system to a research assistant for transcribing. The research assistant removed personally-identifying information from the transcripts. The research coordinator checked each transcript for accuracy by listening to the audio recordings.

### Data analysis

All interview transcripts were uploaded to ATLAS.ti (version 9.1.0), a qualitative data management program [29]. The research coordinator (Author HM) and research team member (Author LJ) read and reread the transcripts for familiarization and then each coded three transcripts. The preliminary codes were reviewed together, and revisions were made. The revised codes were then discussed with the larger research team. After discussing the codes, the transcripts were coded using the agreed upon coding structure. A summary of the coded data and developing themes were reviewed with the research team. Authors HM and LJ further refined themes from the coded data using the analytic techniques of comparing and contrasting within and between key concepts. The full interview transcripts were read and reread to ensure the developing themes captured the context of the interview.

To support the trustworthiness of findings, per Lincoln and Guba (1985), all members of the research team were involved in discussing the preliminary codes and findings [30]. Many of these team members had several years of experience working with PWUS and some had experience working in a treatment setting. We have also provided a detailed description of the methods and study context, as well as participant sociodemographic data, to support transferability of findings.

## Results

The 14 participants were from all four provinces and included directors as well as physicians. Some participants spoke about both OAT and detox programs, and some participants spoke only about one or the other given their knowledge and experiences of the programs.

Most participants identified as women (n = 12), and all as white (n = 14). Most were between the ages of 40–49 years (n = 10). Many had spent 4 years or less working in their current position (n = 8) and many (n = 9) lived in an urban area. See Table 1 for participants’ self-reported sociodemographic information.

**Table 1:** Sociodemographic information (n = 14).

Characteristic	n (n = 14)	%
*Gender Identity*		
Women	12	85.7
Men	2	14.3
*Racial Identity*		
White	14	100.0
*Age Range*		
19−29	--	--
30−39	2	14.3
40−49	10	71.4
50−59	--	--
60−69	2	14.3
*Years Working in Current Position*		
Less than 1 year	2	14.3
1−4 years	6	42.8
5−9 years	2	14.3
10 years or more	4	28.6
*Live in Urban Area?*		
Yes	9	64.3
No	5	35.7

Self-reported sociodemographic information of directors and physicians working at government-funded drug treatment programs in Atlantic Canada (n = 14).

Through an analysis of the interviews, four key themes were developed. Quotes are identified by Site (e.g., A), and participant number (e.g., P1).

### Theme 1: It’s not always easy to access and stay in treatment

A number of participants perceived PWUS as having different emotions when seeking treatment including hope as they [PWUS] were doing something that they believed would help them (Site D, P3), “some degree of happiness or excitement” about getting treatment (Site A, P3), “embarrassment around having a substance use disorder” (Site C, P3), and in some instances a “boatload of trauma” (Site C, P4). Participants noted that various features of programs were not helpful to PWUS at a time when they were seeking treatment including provider stigma, needing a phone to contact a program or receive calls from a program, and wait times. A couple of participants suggested that they believed frustration was what many PWUS felt when they had to wait for treatment, and speaking about PWUS trying to access detox, one participant stated that, *“Sometimes they feel that the wait is a little bit too long…If you talk about the emotions, it’s more about frustration. That’s what we see [among some PWUS]”* (Site C, P1). Not being able to smoke cigarettes on the premises of a treatment program was also noted as a barrier to treatment, and one participant indicated that some PWUS leave treatment if they cannot smoke.

Travel for treatment, particularly for PWUS who must travel daily for OAT, was highlighted by many participants as not helpful or a barrier to retention. One participant commented that, *“I …know of clients who some days have to walk for extended periods of time to get their medication”* (Site C, P3), and another participant stated that, *“And we’ve had in the province [PWUS who] have to come off OAT solely for the reason that they cannot find a viable solution in getting to the pharmacy to get their prescription and get their dose. So it’s been a challenge”* (Site D, P3). According to yet another participant, treatment programs in their province tended to be centrally located and “the rural nature of the province” makes it challenging for those living outside of this area such that transportation “is definitely an issue” (Site B, P1). Travel during poor weather conditions was further noted as a barrier as was the cost of transportation.

OAT dispensing fees are covered for PWUS who are eligible for government income assistance, but some individuals must pay the fees and such costs were identified as not helpful by one participant who stated, **“***It’s kind of that mid person, you know, not the high income earner but the mid earner, who really struggles with the dispensing costs. They’re pretty expensive. So sometimes that certainly can be a barrier”* (Site A, P2). Pharmacies that have limited hours of operation were further cited as creating challenges to retention for some including those who do not have flexible work hours and the pharmacy is closed during times when they need to access treatment. One participant commented that, “*If your pharmacy’s not open, you can only hope that you get take-home dose. If not, sometimes we’ve had someone miss a dose every week”* (Site D, P3).

For PWUS with children, not having childcare was identified as very challenging. A participant noted that some women do not go to detox because there is no one to care for their child(ren) (Site C, P5), and a couple of participants indicated that sometimes women with children miss their appointments because of challenges related to traveling with their child(ren). As one participant stated, “*There’s just another layer of added complexity there in terms of them [women with kids] getting to the clinic to get the prescription and then having to go to a pharmacy”* (Site C, P3). Some PWUS are fearful of child protective services, and this was noted as affecting access.

### Theme 2: Program features that can help with access and retention

When asked about what helped with access, quick access or short wait times were highlighted. A participant commented that wait times at their program had recently been almost eliminated because they were able to expand their program with increasing resources, and a couple of other participants spoke about a provincial intake program in their province that helped with access as PWUS phoned a centralized number, and were then provided with treatment options (e.g., OAT, detox) depending on their needs.

A number of participants also stated that being quickly responsive to a client’s individual needs helped with retention, and a few participants pointed to the importance of an individualized approach as helping with retention. Moving a client to another clinic for treatment “for a little bit” was one example of an individualized approach if there were challenging behaviours (Site A, P2), or having some “restrictions” but not dismissing someone from a program. One participant commented as follows:

*…There’s all kinds of things that can come up and the first response can’t be to kick the person out of the program…I’m not saying that we don’t put restrictions on people sometimes. Because sometimes that’s necessary. I mean we have to think of the safety of the other clients and staff as well…They [PWUS] aren’t kicked out of the program but they are like, ‘Okay you come, you get your medication, you leave. You can’t hang out here’* (Site D, P1).

Other features of treatment programs that were highlighted as helpful to access and retention included caring, non-judgmental staff who had good rapport or a connection with PWUS. One participant maintained that, “*You need to make a connection. You need to sit down with them, even if they’ve been here ten times, it doesn’t make a difference….have a conversation with them. Maybe they need something to eat* …” (Site C, P6). Having social workers was further noted as important in supporting retention as social workers linked PWUS to a variety of government or community services such as income assistance programs, housing supports, or counseling. Various ‘groups’ and activities were also highlighted as helpful for retention, and one participant indicated that at their program they have had “gardening groups” as well as “self-care groups [and] meditation groups” (Site A, P2). Pharmacies that remained open even during adverse weather conditions was further noted by one participant as very helpful as it ensured access to treatment even when other pharmacies were closed.

Supports and services related to the needs of PWUS in various socio-economic situations were discussed by a few participants as helpful. For example, a participant spoke about an early morning and evening OAT clinic for PWUS who had jobs or were attending school, and a couple of participants noted practices to help with phone access (e.g., pharmacies allowing PWUS to use the pharmacy phone to contact a provider, or a treatment program providing access to a phone for a period of time). A few participants mentioned help with transportation, and a participant stated that at their treatment program they sometimes provided a bus ticket to PWUS. Supports for those who smoke cigarettes were also mentioned. One participant stated that at their program there was a smoking area, and a couple of other participants noted that at their program they offered nicotine replacement (e.g., patches).

Informal supports were further highlighted as helpful to retention, and a participant reported that when they first started their program “a couple of the moms and one of the community advocates” came and sat in the waiting room to help provide support (Site D, P1). Peer supports were also noted as helpful, and a participant commented that the “power of the waiting room conversations” where there are peers who are “there for the same reason” helped with OAT retention (Site C, P4).

### Theme 3: Changes during COVID: sometimes helpful, sometimes not

A few participants spoke about changes in policies and/or practices during COVID which helped to reduce some of the challenges to access and retention at their program. For example, in some places there was a move to online or phone appointments which helped to reduce wait times and travel requirements. Medication delivery from a pharmacy when a PWUS was isolating due to COVID was also mentioned as a helpful practice. According to one participant, prior to some of the changes in practice due to COVID, some PWUS living in rural areas might have to drive a couple of hours to access a pharmacy. This participant stated that:

*But I think with COVID, we have been forced to be creative. And a lot of the virtual and telehealth options are now a possibility for those individuals [who previously had to travel hours to a pharmacy]* (Site D, P3).

A couple of participants noted that during COVID the treatment program did not conduct urinalysis as often, and some participants also mentioned that there was quicker or greater access to “carries” or take-home doses of OAT. Both of these changes would have meant reduced travel requirements. Commenting on changes in frequency of “urine drug screens” that happened because of COVID, one participant stated that:

… *it’s always been a pretty closed system in terms of going to the pharmacy every day and having urine drug screens done. A paternalistic approach based on decades of data that has informed a given level of practice. And since the pandemic urine drug screens have dropped… and it’s had no effect on negative outcomes…I think if anything for the individual patient or client access has improved* (Site A, P3).

A couple of participants indicated that although the move to phone and virtual care was helpful for some PWUS it did not help all PWUS because of the costs associated with phones and the internet, and internet connectivity issues especially in rural places. As one participant commented, phone contact occurred quite a lot during COVID but, “*There was a loss of contact with the clinic for some folks who were not available by phone or couldn’t afford a phone or didn’t have any minutes on their phone. And so that was problematic”* (Site C, P4). Limited or reduced detox beds during COVID because of the need for physical distancing was also noted as not helpful, and one participant commented that during COVID “groups” were impacted, “because of safety and social distancing*”* and the groups were missed given their role in supporting retention (Site D, P3).

### Theme 4: What is needed: better linkages with mental health, some practice changes, and more resources

A few participants highlighted (either explicitly or implicitly) changes that they believed are needed to further support access and retention including better linkages between addiction and mental health services. Speaking about detox programs, one participant suggested that there should be a “combination of mental health and addiction” programs or services on the “same unit” (Site C, P1). This participant stated that “*…a lot of the time they’re [PWUS] not being admitted on the psych unit because they [staff] think it’s an addiction problem first”* (Site C, P1).

A few participants suggested that in some programs there is a need for practice changes. One participant commented that there should be no wait time for a detox bed, and another stated that*, “It would be nice to have something to access 24 hours a day, 7 days a week*” (Site C, P1). Having the treatment option of long-lasting injectable OAT was noted as important because it would mean that that PWUS would not have to make daily trips to the pharmacy. According to a couple of participants there should be more “follow up” or supports in place when individuals leave treatment, and one participant maintained that “there’s not a lot of follow-up” when someone leaves treatment, and this is a current “gap” in services (Site C, P5). Addressing stigma was also commented upon, and one participant indicated that they had seen gains in the reduction of stigma within the area of mental health in recent years and they hoped to “see it on the substance use disorder side…” (Site C, P3). Hiring peers to engage in peer supports was further suggested as a practice that would help with retention.

A couple of participants argued that there was a need for more resources to help with treatment access. Speaking about OAT, one participant stated that, “*I think we’re underfunded and under resourced”* (Site C, P3), and another commented, “*We don’t have the resources to keep up with it [need for addiction services], really”* (Site D, P2). According to one participant, in their community there were too few physicians to do detox “admissions” which had an impact on wait times (Site A, P1). Having more community-based OAT clinics (as necessary) was recommended by another participant (Site C, P4), and increased resources would assist with implementing such clinics.

## Discussion

This paper highlights government-funded treatment program directors’ and physicians’ perspectives on how program features inform treatment access and retention in Atlantic Canada. Participants identified a few features that helped with access, such as quick and easy program intake and individualized treatment, as well as some features that did not help, including wait times, travel time and transportation challenges. Some of these not helpful features have been reported by researchers in other parts of Canada and the United States based on research conducted years ago [31,32]. More recent research in Canada, including our previous research with PWUS, family members and community-based organization staff, has identified some of the same helpful and not helpful features [17,23,24]. For instance, across all phases of our research, quick and easy access was identified as a helpful feature. Participants in all phases of our research also highlighted similar features that were not helpful for access and retention, including location of programs, limited transportation, and limited operating hours. Findings from this paper adds the perspectives of directors and physicians working in treatment programs in Atlantic Canada, and much of what they have said supports the current body of literature on features of treatment programs that help or do not help with access and retention. Despite the relative consistency of findings from this research with previous phases of research, we also identified new key findings on the impact of COVID-19 on treatment program access in Atlantic Canada which adds to the literature. Although findings are from one region in Canada, they may be relevant to other small urban centres.

During COVID-19, many treatment programs in Canada and the United States implemented changes in response to public health requirements [33–35]. In some places, for example, mandatory physical distancing was implemented to prevent the spread of COVID-19 resulted in moving at least some in-person services to virtual services [35]. Some participants in our research reported that a few of these changes also occurred in their programs, such as moving to virtual appointments and reducing the frequency of required urinalysis. Our research reported, and other research studies have similarly reported, that some of these changes helped, and others did not with access to and retention in treatment [36–38].

Many of the changes made during COVID-19 were implemented rapidly to respond to evolving public health measures and to address the need to provide treatment in the context of COVID. This highlights that relatively speedy changes are possible although external pressures may be necessary to ensure such changes. Even though these changes were made to prevent the spread of COVID-19, at least some should be continued as they have been found to help with access to treatment. However, we are also cognizant that these changes did not equitably support access to treatment for all PWUS given differences in socioeconomic status. For example, although a few participants in our study believed that the switch to virtual services helped many PWUS with access by removing transportation-related barriers, some participants also believed that the cost of engaging with virtual services, such as the cost of mobile phone minutes and data, did not help some PWUS with access. Therefore, in addition to continuing some of the program changes made during COVID-19, additional changes will be needed to equitably support access for all PWUS.

Beyond some of the changes that occurred during COVID-19, some participants highlighted other changes that they believed are needed to help improve access and retention in their programs. One participant suggested better linkages between mental health supports and addiction treatment, and a few participants suggested reduced wait times. Since our study, a mental health and addictions emergency department has opened in Prince Edward Island [39]. This 24/7 unit provides ‘one-stop’ access to readily available mental health and addictions services, including treatment plans and linkages to community supports. It indicates an interest in integrating mental health and addiction services and to have the services accessible 24/7, but it is not clear exactly what services are available. A couple of participants in the study we have reported on in this paper also suggested the need for peer support embedded in treatment programs. Peer support refers to people with lived experience of substance use sharing their experiences and providing support to individuals in treatment [40]. Implementation of peer support within the treatment system varies with some treatment services offering training and certifications for peer support workers [41,42]. Delivery of peer support can also vary. One clinic in rural and remote British Columbia provides peer-led OAT medication delivery which has helped with access to and retention in treatment by addressing transportation challenges and providing peer support [43]. There have also been changes to another feature that was identified as not helpful to access in this paper. In British Columbia, PWUS who previously had to pay for OAT medications no longer need to pay because the cost of medications is now “covered through Plan Z, the Province’s universal coverage plan, providing full coverage for B.C. residents with an active medical services plan” [44]. Finding champions within the health system may be key to building momentum, gaining ‘buy-in’ from leadership, and navigating challenges to implementation [45,46].

Several participants pointed to the need for more resources for addiction treatment, and this is not surprising given that in Canada, evidence-based treatment programs have been vastly underfunded in comparison to other interventions that are a part of the Canadian Drugs and Substances Strategy. For instance, between 2017−18 and 2021−22, Canada spent nearly $742 million to address substance use and of that $742 million, $432.69 million (58%) was spent on enforcement and only $93.06 million (13%) on treatment [47]. Given that substance use is a pressing issue in Canada, providing adequate funding for evidence-based treatment is essential. Considering that changes were rapidly made to treatment programs during COVID-19, the healthcare system has the capacity to adapt and enhance programs to meet the needs of PWUS, but it must be adequately resourced by both federal and provincial governments.

### Limitations and future research

Interviews with directors and physicians occurred during the COVID-19 pandemic when healthcare professionals, including individuals working in treatment programs, were experiencing workload pressures. Some individuals eligible to participate in our study may have wanted to participate but been unable to given their workload. If this is the case, not hearing the perspectives of these individuals is a potential limitation of our study.

Our research focused on the perspectives of directors and physicians working in government-funded treatment programs delivered through provincial health authorities in Atlantic Canada. Other individuals working in the treatment sector, including in pharmacies, family medicine practices, and private treatment programs, might have different perspectives which are not part of this paper. Further research is needed to understand the perspectives of individuals working in these areas. Future research could also examine organizational and community-level factors for implementing policy and program changes to support access to treatment programs in the region.

## Conclusions

Physicians and directors identified a range of treatment program features that were not helpful for accessing treatment, including travel, OAT dispensing fees, and limited pharmacy operating hours. However, they also noted some features that helped with access, such as quick access and individualized treatment. Some changes to programs were implemented due to COVID-19 and these changes were viewed by participants as being helpful for ensuring access for some PWUS and not as helpful for others, largely due to differences in socioeconomic status. Changes that were helpful for ensuring access should be maintained and additional changes should be made to ensure equitable access for all PWUS. Increased resources must be invested to support these changes and, ultimately, support access to and retention in treatment.

## Supporting information

S1 Appendix Semi-structured interview guide. Semi-structure interview guide used with physicians and directors of publicly-funded treatment programs in Atlantic Canada.
(DOCX)
