# Supplementary material for: Features of drug addiction treatment programs in Atlantic Canada that help (or not) with access and retention: A qualitative study
Source: PLoS One. 2025 Aug 4;20(8):e0328524. doi: 10.1371/journal.pone.0328524 (PMC12321136; doi:10.1371/journal.pone.0328524)
Supplement: S1 Appendix — Semi-structure interview guide used with physicians and directors of publicly-funded treatment programs in Atlantic Canada. (DOCX) [file pone.0328524.s001.docx]

**Interview Guide**

**PHASE 3 – Physicians and/or Directors of publicly-funded drug addiction treatment programs**

***Preamble***

I would like to know about **your perceptions** of your clients’ experiences when accessing/staying in/leaving publicly-funded drug addiction treatment programs. I am interested in two types of public programs: 1: opioid assisted treatment (e.g., methadone maintenance treatment, suboxone, Kadian); and 2: detox or withdrawal programs.

I will ask you about each type of program. We are interested in your perspectives on programs and policies in the **past 2 years or so, including any program practices or policies that might act as barriers and/or facilitators to access and retention.** There are no right or wrong answers.

1. **Opioid Assisted Treatment Program (For those participants providing OAT in their program)**

*Points of Access:*  ***Potential Questions/Probes***

| **Reasons for accessing/not accessing** | 1. Can you tell me about **your perceptions** of the experiences your clients have had in the past two years accessing opioid assisted treatment (i.e. methadone maintenance treatment, suboxone, Kadian)?  **Probes:**  a) Where was the program?  b) Why do you perceive that they accessed this  program?  c) If they did not access a program, why do you perceive that they did not?  i) Did they have fears related to child protection?  ii) Did they have difficulties getting an appointment? (e.g. had to phone, long wait times)? |
| --- | --- |

| **Trying to get in** | 2a Can you tell me **about any program policies and/or practices that you perceived were helpful** for your clients when trying to get in?  **Probes:**  a) Was there program support for transportation or  childcare?  b) Did the program let them in quickly?  c) Could they keep smoking while in the program?  d) Could they keep using some drugs while in  program?  e) Was there supportive program staff?  f) What other program rules helped them get into the  program?  2b. What do you perceive was the rationale behind the  various practices or policies?  **Probe:**  a) Was this policy/practice impacted by COVID-19?  That is, were there any changes to this  practice/policy due to COVID-19. Can you  explain?  3a Can you tell me **about any program policies and/or practices that you perceived were not helpful** for your clients when trying to get in?  **Probes:**  a) Was there a long waiting time to get in?  b) Did they need to phone each day to check for a spot?  c) Did the program respond to their continued drug  use/return to drug use?  3b. What do you perceive was the rationale behind the various practices or policies?  **Probe:**  a) Was this policy/practice impacted by COVID-19?  That is, were there any changes to this  practice/policy due to COVID-19. Can you  explain?  4. What do **you perceive** was the **impact on your clients** when they were trying to get into the program? **Why** do you think they felt that way?  **Probes:**  a) Did they have feelings of frustration/madness/  sadness/disappointment or hopelessness?  b) Did they feel in control or not in control?  c) Did they have any fear of Child Protection Services  taking away children?  d) Did it have no real impact on their feelings?  5. Did **you perceive** any **changes to your clients’ drug use, safer drug use or other related behaviours** when they were trying to get into the program? **Why** do you think this happened?  **Probes:**  a) Did they continue or discontinue drug use?  b) Did they experience a change in how they were  using (e.g., smoking and not using injection drugs)?  c) Did they use in safer ways or use less safely? |
| --- | --- |
| **Getting in and not staying (voluntary or involuntary leave)** | I would now like to ask you a few questions about how the program policies and practices may have influenced clients who **were in the program but did not stay**. Or, in other words left either voluntarily or involuntarily.  6a. Can you tell me **about any program policies and/or practices that you perceived were helpful** for your clients when they were **in** the program, even though they may not have stayed?  **Probes:**  a) Could they keep smoking?  b) Could they keep using some drugs?  c) Was there supportive staff?  d) Did they have access to HIV testing?  6b. What do you perceive was the rationale behind the various practices or policies?  **Probe:**  a) Was this policy/practice impacted by COVID-19?  That is, were there any changes to this  practice/policy due to COVID-19. Can you  explain?  7a. Can you tell me **about any program policies and/or practices that you perceived were not helpful** for your clients when they were in the program, and may have influenced them leaving the program?  **Probes:**  a) Were they unable to smoke or use other  substances?  b) Did they have difficulty following rules?  c) Did they have to leave as a response to continued  drug use/return to drug use?  d) Was there a lack of program content?  7b. What do you perceive was the rationale behind the various practices or policies?  **Probe:**  a) Was this policy/practice impacted by COVID-19?  That is, were there any changes to this  practice/policy due to COVID-19. Can you  explain?  7c. Do you perceive that these were the reasons that they  did not stay in treatment?    8. How do **you perceive** **your clients felt about the program practices and policies** when they were in the program, even though they did not stay (e.g. voluntary or involuntary leave)? **Why** do you think they felt that way?  **Probes:**  a) Did they have feelings of frustration/madness/  sadness/disappointment or hopelessness?  b) Did they feel in control or not in control?  c) Did they have any fear of Child Protection Services  taking away children?  d) Did it have no real impact on their feelings?    9. Did **you perceive** any **changes to your clients’ drug use, safer drug use or other related behaviours** when they were in the program, even though they did not stay? **Why** do you think this happened?  **Probes:**  a) Did they continue or discontinue drug use?  b) Did they experience a change in how they were  using (e.g. smoking and not using injection drugs)?  c) Did they use in safer way or use less?  10. Can you tell me what **you perceive** it was like **for your clients when they left** the program?  **Probes:**  a) Did they have support systems in place?  b) Did they have access to safe supplies? |
| **Staying in the program** | 11a. Can you tell me **about any program practices or policies that you perceive were helpful** for your clients to help them **stay in the program?**  **Probes:**  a) Could they keep smoking?  b) Could they keep using some drugs?  c) Was there supportive staff?  d) Did they have access to HIV testing?  11b. What do you perceive was the rationale behind the various practices or policies?  **Probe:**  a) Was this policy/practice impacted by COVID-19?  That is, were there any changes to this  practice/policy due to COVID-19. Can you  explain?  12a. Can you tell me **about any program policies/practices that you perceive were not helpful** for your clients when trying to stay in the program?  **Probes:**  a) Were they unable to smoke or use other  substances?  b) Did they have difficulty following rules?  c) Did they have to leave as a response to  continued drug use/return to drug use?  d) Was there a lack of program content?  e) Did they have to undergo extensive urinalysis?  f) Were they penalized for missing appointments?  g) Was the program’s approach to dosing or a  change in dosing unhelpful?  h) Did they have difficulties accessing carries? Did  they lose their carry privileges?  i) Did they perceive staff as being unhelpful or  unsupportive?  12b. What do you perceive was the rationale behind the various practices or policies?  **Probe:**  a) Was this policy/practice impacted by COVID-19?  That is, were there any changes to this  practice/policy due to COVID-19. Can you  explain?  12c. Do you perceive that these were the reasons that they  did not stay?    13. How do you perceive it **your clients felt** when they stayed in the program? **Why** do you think they felt that way?  **Probes:**  a) Did they have feelings of frustration/madness/  sadness/disappointment or hopelessness?  Happiness/pride/hope?  b) Did they feel in control or not in control?  c) Did they have any fear of Child Protection Services  taking away children?  d) Did it have no real impact on their feelings?  14. Did **you perceive** any **changes to your clients’ drug use, safer drug use or other related behaviours** when they stayed in the program? **Why** do you think this happened?  **Probes:**  a) Did they continue or discontinue drug use?  b) Did they experience a change in how they were  using (e.g. smoking and not using injection drugs)?  c) Did they use in safer way or use less?    15. Can you tell me what **you perceive** it was like or your clients **when they left** the program?  **Probes:**  a) Did they have support systems in place?  b) Did they have access to safe supplies? |

16. Do you have any other perceptions of your clients accessing opioid assisted treatment programs that you would like to share?

1. **Detox/Withdrawal Program (For those participants who work in detoxification of withdrawal programs)**

*Points of Access:*  ***Potential Questions/Probes***

| **Reasons for accessing/not accessing** | 1. Can you tell me about **your perceptions** of the experiences your clients have had in the past two years accessing detox/withdrawal programs?  **Probes:**  a) Where was the program?  b) Why do you perceive that they accessed this program?  c) If they did not access a program, why do you perceive that they did not?  i) Did they have fears related to child protection?  ii) Did they have difficulties getting an appointment?  (e.g. had to phone, long wait times)? |
| --- | --- |

| **Trying to Get In** | 2a Can you tell me **about any program policies and/or practices that you perceived were helpful** for your clients when trying to get in?  **Probes:**  a) Was there program support for transportation or  childcare?  b) Did the program let them in quickly?  c) Could they keep smoking in the program?  d) Could they keep using some drugs in program?  e) Was there supportive program staff?  f) What other program rules helped them get into the  program?  2b. What do you perceive was the rationale behind these various practices or policies?  **Probe:**  a) Was this policy/practice impacted by COVID-19?  That is, were there any changes to this  practice/policy due to COVID-19. Can you  explain?  3a. Can you tell me **about any program policies and/or practices that you perceived were not helpful** for your clients when trying to get in?  **Probes:**  a) Was there a long waiting time to get in?  b) Did they need to phone each day to check for a spot?  c) Did the program respond to their continued drug  use/return to drug use?  d) Was their cell phone use restricted?  3b. What do you perceive was the rationale behind these various practices or policies?  **Probe:**  a) Was this policy/practice impacted by COVID-19?  That is, were there any changes to this  practice/policy due to COVID-19. Can you  explain?    4. What do **you perceive was the impact on your clients** when they were trying to get into the program? **Why** do you think they felt that way?  **Probes:**  a) Did they have feelings of frustration/madness/  sadness/disappointment or hopelessness?  b) Did they feel in control or not in control?  c) Did they have any fear of Child Protection Services  taking away children?  d) Did it have no real impact on their feelings?  5. Did **you perceive** any **changes to your clients’ drug use, safer drug use or other related behaviours** when they were trying to get into the program? **Why** do you think this happened?  **Probes:**  a) Did they continue or discontinue drug use?  b) Did they experience a change in how they were  using (e.g. smoking and not using injection drugs)?  c) Did they use in safer way or use less? |
| --- | --- |
| **Getting In and Not Staying (Voluntary or involuntary leave)** | I would now like to ask you a few questions about how the program policies and practices may have influenced clients who were in the program but did not stay. Or, in other words left either voluntarily or involuntarily.  6a. Can you tell me **about any program policies and/or practices that you perceived were helpful** for your clients when they were **in** the program, even though they may not have stayed?  **Probes:**  a) Could they keep smoking?  b) Could they keep using some drugs?  c) Was there supportive staff?  d) Did they have access to HIV testing?  e) Could they use their cell phone while in the  program?  6b. What do you perceive was the rationale behind these various practices or policies?  **Probe:**  a) Was this policy/practice impacted by COVID-19?  That is, were there any changes to this  practice/policy due to COVID-19. Can you  explain?  7a. Can you tell me **about any program policies and/or practices that you perceived were not helpful** for your clients when they were in the program, and may have influenced them leaving the program?  **Probes:**  a) Were they unable to smoke or use other  substances?  b) Did they have difficulty following rules?  c) Did they have to leave as a response to continued  drug use/return to drug use?  d) Was there a lack of program content?  e) Was their cell phone use restricted?  7b. What do you perceive was the rationale behind these various practices or policies?  **Probe:**  a) Was this policy/practice impacted by COVID-19?  That is, were there any changes to this  practice/policy due to COVID-19. Can you  explain?  7c. Do you perceive that these were the reasons that they  did not stay?  8. How do **you perceive** **your clients felt about the program practices and policies** when they were in the program, even though they did not stay (e.g. voluntary or involuntary leave)? **Why** do you think they felt that way?  **Probes:**  a) Did they have feelings of frustration/madness/  sadness/disappointment or hopelessness?  b) Did they feel in control or not in control?  c) Did they have any fear of Child Protection Services  taking away children?  d) Did it have no real impact on their feelings?    9. Did **you perceive** any **changes to your clients drug use, safer drug use or other related behaviours** when they were in the program, even though they did not stay? **Why** do you think this happened?  **Probes:**  a) Did they continue or discontinue drug use?  b) Did they experience a change in how they were  using (e.g. smoking and not using injection drugs)?  c) Did they use in safer way or use less?    10. Can you tell me what **you perceive** it was like **for your clients when they left** the program?  **Probes:**  a) Did they have support systems in place?  b) Did they have access to safe supplies? |
| **Staying in the program** | 11a. Can you tell me **about any program practices or policies that you perceive were helpful** for your clients to help them **stay in the program?**  **Probes:**  a) Could they keep smoking?  b) Could they keep using some drugs?  c) Was there supportive staff?  d) Did they have access to HIV testing?  e) Did they have access to counselling?  f) Could they use their cell phone?  11b. What do you perceive was the rationale behind the various practices or policies?  **Probe:**  a) Was this policy/practice impacted by COVID-19?  That is, were there any changes to this  practice/policy due to COVID-19. Can you  explain?  12a. Can you tell me **about any program policies/practices that you perceive were not helpful** for your clients when trying to stay in the program?  **Probes:**  a) Were they unable to smoke or use other substances?  b) Did they have difficulty following rules?  c) Did they have to leave as a response to continued  drug use/return to drug use?  d) Was there a lack of program content?  e) Did they perceive staff as being unhelpful or  unsupportive?  12b. What do you perceive was the rationale behind the various practices or policies?  **Probe:**  a) Was this policy/practice impacted by COVID-19?  That is, were there any changes to this  practice/policy due to COVID-19. Can you  explain?  12c. Do you perceive that these were the reasons that they  did not stay?    13. How do **you perceive** it **your clients felt** when they stayed in the program? **Why** do you think they felt that way?  **Probes:**  a) Did they have feelings of frustration/madness/  sadness/disappointment or hopelessness?  Happiness/pride/hope?  b) Did they feel in control or not in control?  c) Did they have any fear of Child Protection Services  taking away children?  d) Did it have no real impact on their feelings?  14. Did **you perceive** any **changes to your clients’ drug use, safer drug use or other related behaviours** when they stayed in the program? **Why** do you think this happened?  **Probes:**  a) Did they continue or discontinue drug use?  b) Did they experience a change in how they were  using (e.g. smoking and not using injection drugs)?  c) Did they use in safer way or use less?    15. Can you tell me what **you perceive** it was like or your clients **when they left** the program?  **Probes:**  a) Did they have support systems in place?  b) Did they have access to safe supplies? |

16. Do you have any other perceptions on your clients’ experiences with detox or withdrawal programs that you would like to share?

**3. Additional Thoughts**

Based on your experiences, is there anything else you want to tell me about your clients’ experiences with publicly-funded drug addiction treatment programs? Based on your perceptions, how might drug addiction treatment programs be improved to meet the needs of people who use substances?

**4. Demographics**

Before we end the interview, I would like to ask you a few questions about your background.

A: Can you tell me how many years you have been working in your current position?

Answer: ___________

B: Can you tell me what age range you fall within?

20-29 years ______

30-39 years ______

40-49 years ______

50-59 years ______

60-69 years _______

C: Can you tell me your gender? (e.g. male, female, trans, 2-spirited, etc?)

Answer: ______________

D. Can you tell me your ethnicity? (e.g. Caucasian, Black, Indigenous)

Answer: _____________

D: Do you live in a city (e.g., Halifax, St. John’s, Moncton) or outside of a city? If outside, where?

City ________

Outside ________
